# Neuroprotective Effects of 7, 8-dihydroxyflavone on Midbrain Dopaminergic Neurons in MPP^+^-treated Monkeys

**DOI:** 10.1038/srep34339

**Published:** 2016-10-12

**Authors:** Jingjing He, Zheng Xiang, Xiaoqing Zhu, Zongyong Ai, Jingsong Shen, Tianzhuang Huang, Liegang Liu, Weizhi Ji, Tianqing Li

**Affiliations:** 1 Yunnan Key Laboratory of Primate Biomedical Research; Institute of Primate Translational Medicine, Kunming University of Science and Technology, Kunming, 650500, Yunnan, China; 2 Department of Nutrition and Food Hygiene, Hubei Key Laboratory of Food Nutrition and Safety, School of Public Health, Tongji Medical College, Huazhong University of Science and Technology, 13 Hangkong Road, Wuhan 430030, China

## Abstract

Parkinson’s disease (PD) is one common neurodegenerative disease caused by a significant loss of midbrain dopaminergic neurons. Previous reports showed that 7, 8- dihydroxyflavone (7, 8-DHF) as a potent TrkB agonist can mimic BDNF and play neuroprotective roles for mouse dopaminergic neurons. Nonetheless, the safety and neuroprotective effects are unclear in monkey models of PD. Here, we find that 7, 8-DHF could be absorbed and metabolized into 7-hydroxy-8-methoxyflavone through oral administration in monkeys. The half-life time of 7, 8-DHF in monkey plasma is about 4–8 hrs. Furthermore, these monkeys maintain health state throughout the course of seven-month treatments of 7, 8-DHF (30 mg/kg/day). Importantly, 7, 8-DHF treatments can prevent the progressive degeneration of midbrain dopaminergic neurons by attenuating neurotoxic effects of MPP^+^ and display strong neuroprotective effects in monkeys. Our study demonstrates that this promising small molecule may be transited into a clinical useful pharmacological agent.

Parkinson’s disease (PD) is one common neurodegenerative disease with typical motor symptoms including bradykinesia, postural instability, rigidity and tremor, and characteristic pathological change, resulting from dopaminergic neuron death in the substantia nigra pars compacta (SNc) of midbrain^1^. Over the past few decades, there were major advances on neurological treatments of PD including pharmacological treatments, physical or behavioral therapy, deep brain stimulation (DBS) and noninvasive stimulation approaches^2^,^3^. However, these treatments are temporary symptomatic relief; so far, no treatment has delayed the onset and/or slowed the progression of this disease^4^.

BDNF regulates the development of several classes of neurons and mediates their maintenance and survival. BDNF binding to TrkB triggers its dimerization and autophosphorylation of tyrosine residues in its intracellular domain, and displays potent neurotrophic actions on neuronal populations involved in several neurodegenerative diseases including Parkinson disease (PD)^5^. However, the outcomes of several clinical trials using recombinant BDNF are disappointing due to the poor pharmacokinetic properties^6^. Previous studies showed that 7, 8-dihydroxyflavone (7, 8-DHF), as a potent and selective agonist of TrkB, can mimic BDNF, is orally bioactive and penetrates brain blood barrier^5^. Furthermore, 7, 8-DHF treatments attenuate the neurotoxic effects of MPTP as measured by preservation of tyrosine hydroxylase expression and suppression of activated caspase-3, implying that the TrkB agonist has neuroprotective potential for dopaminergic neurons in the SNc of mouse PD^5^.

The genomics, brain anatomy and neuronal circuitry are significantly different between primates (including human) and rodents. The MPTP-treated monkey is considered as the “gold standard” model of parkinsonism because of the high similarities in motor behavioral changes and basal ganglia pathophysiology between this model and the human Parkinson’s disease condition^4^,^7^. Thus, non-human primate models of PD induced by MPTP are important for evaluating potential protective efficiency of 7, 8-DHF on dopaminergic neurons in primates before moving into clinical trials to test its efficacy in patients with PD. MPTP selectively targets the dopamine system by dopamine transporter-mediated uptake of the toxic metabolite 1-methyl-4-phenylpyridinium (MPP^+^) in monkeys^8^,^9^,^10^. MPP^+^ has been shown to inhibit complex I and produce free radicals, resulting in the death of the neurons. Recently, a new approach was used to develop Parkinsonian monkeys with intracerebroventricular injections of MPP^+^^11^. This new method avoids the damage of gastrointestinal dopamine neurons induced by MPTP and specifically targets brain dopamine neurons^11^.

Here, we show that 7, 8-DHF could be absorbed and metabolized into 7-hydroxy-8-methoxyflavone through oral administration in monkeys and maintain monkeys in health state throughout the course of seven-month treatments. Importantly, 7, 8-DHF treatment can prevent the progressive degeneration of midbrain dopaminergic neurons induced by MPP^+^ in the monkey model.

## Results

### 7, 8-DHF were absorbed and metabolized into 7-hydroxy-8-methoxyflavone in monkeys

**Figure 1:**
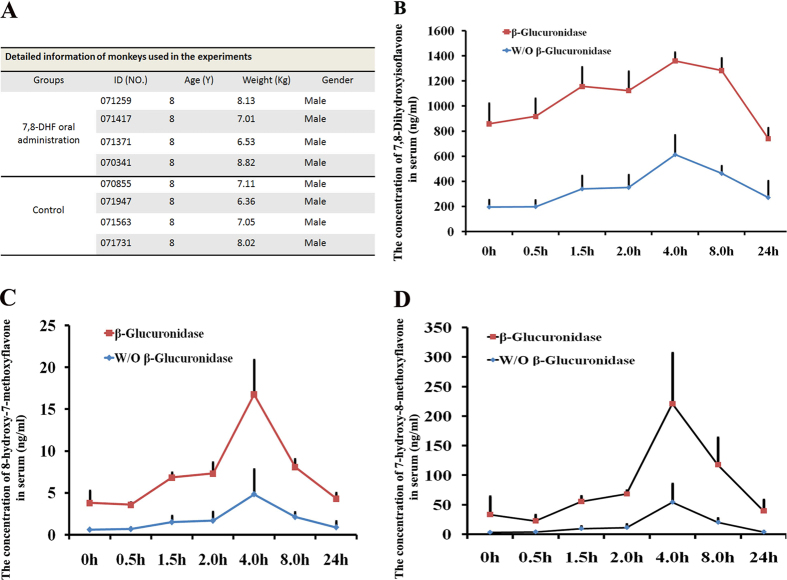
7, 8-DHF were absorbed and metabolized into 7-hydroxy-8-methoxyflavone in monkeys. (**A**) The detailed information of monkeys used in the studies. (**B**) The concentration dynamics of 7, 8-DHF in monkey plasma within 24 hrs after oral administration. (**C,D**) The concentration dynamics of two matebolites, 8-hydroxy-7-methoxyflavone and 7-hydroxy-8-methoxyflavone, of 7, 8-DHF in monkey plasma within 24 hrs after oral administration, respectively. W/O β-Glucuronidase indicated the measures were performed in the condition absence of β-Glucuronidase.

Eight 8-year-old cynomolgus monkeys (Macaca fascicularis) were randomly divided into 7, 8-DHF group and control group (four monkeys each group) to minimize the varieties between animals. The detailed information of monkeys used in the study is shown in Fig. 1A. To evaluate *in vivo* distribution of 7, 8-DHF, 30 mg/kg of dose was orally administrated every day. The blood samples were collected and the concentrations of 7, 8-DHF and its two major metabolites, 8-hydroxy-7-methoxyflavone and 7-hydroxy-8-methoxyflavone, were determined by HPLC in presence or absence of β-glucuronidase, an enzyme that catalyzes breakdown of complex carbohydrates^12^, respectively.

Before examination, the monkeys of 7, 8-DHF group have been treated by 7, 8-DHF for two months. The plasma samples were first collected at the 8:00 am with a 24-hour interval from last oral administration of 7, 8-DHF. 857.43 ± 164.45 ng/ml or 193.77 ± 58.07 ng/ml of 7, 8-DHF (n = 4) were detected when glucuronidase was used or not in the examination, respectively (Fig. 1B). To further evaluate dynamics of 7, 8-DHF and its two major metabolites within 24 hrs a day, the plasma samples from eight monkeys (4 for control group; 4 for 7, 8-DHF group) at 0.5-, 1.5-, 2.0-, 4.0-, 8.0- and 24- hours after oral administration were collected for examination, respectively. 7, 8-DHF treatment leaded to its significant increases at 1.5 hrs in the circulation system with a peak at 4 hrs (Fig. 1B). It sustained in the circulation for 8–24 hrs with a peak plateau at 4–8 hrs, which is extremely encouraging for the long half-life (about 4–8 hrs) in the primates (Fig. 1B). This compound could be methylated on both 7- and 8-hydroxy positions (Fig. 1C,D). The concentrations of moth mono-methylated matebolites peaked at the 4th hour in serum plasma, correlating with 7, 8-DHF. Notably, 8-methylated matebolite is the major metabolite, which is a 12-fold higher than another 7-methylated metabolite (Fig. 1C,D). These data are consistent with previous findings that both metabolites are bioactive in activating TrkB with 7-hydroxy stronger than 8-hydroxy metabolites in mouse^13^. Nonetheless, the 7, 8-DHF concentrations in the plasma are approximately 10 folds higher than those of the methylated metabolite. The concentrations of 7, 8-DHF and 7-hydroxy-8-methoxyflavone in plasma declined to the initial concentration after 24 hrs (Fig. 1B–D). In contrast, we failed to detect 7, 8-DHF and their metabolites in plasma from the four control monkeys at any time.

To further evaluate 7, 8-DHF and its metabolites *in vivo*, we also examined their presence in the monkey urine and feces. The samples from urine and feces at six consecutive nights were collected, respectively. 7, 8-DHF and 7-hydroxy-8-methoxyflavone were obviously detected in all monkey urine and feces (Figure S1A–S1D). The 7, 8-DHF concentrations in the monkey urine were significantly higher than those in the monkey plasma (Fig. 1B–D and S1A–S1D). Interestingly, high concentration of 7-hydroxy-8-methoxyflavone was also detected in monkey feces, implying that 7, 8-DHF can be metabolized into 8-methylated in the gastrointestinal system. Together, these data imply that 7, 8-DHF was successfully absorbed and metabolized in monkeys.

### Monkeys maintained health state throughout the course of 7, 8-DHF treatments

**Figure 2:**
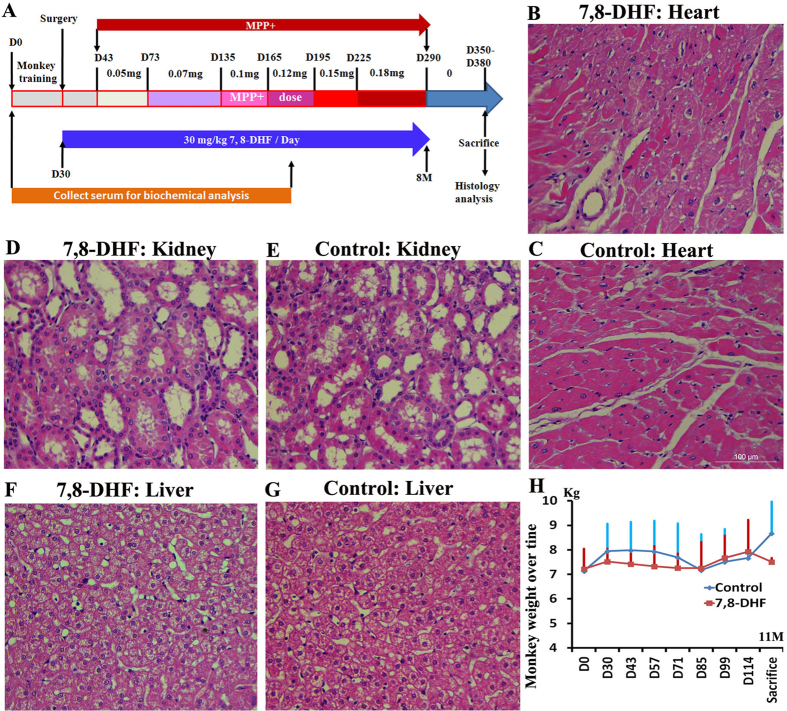
Long-term oral administration of 7, 8-DHF is safe for monkeys. (**A**) Schematic representation of MPP^+^ induction and 7, 8-DHF oral administration. (**B–G**) The representative HE staining of heart, kidney and liver from 7, 8-DHF and control group monkeys, showing that no any abnormal phenotype was observed in the 7, 8-DHF group. (**H**) The weight change of control group and 7, 8-DHF group monkeys over time.

To evaluate possible side effects of 7, 8-DHF for monkeys after long-term treatments, we collected the plasma from 7, 8-DHF and control monkeys every two weeks within nine months and examined their physiological and biochemical parameters, which are presage of hematology, liver, heart, muscle, kidney and pancreas function (Fig. 2A). As expected, long-term oral administrations of 7, 8-DHF (30 mg/kg/day) did not result in abnormal hematology as well as abnormal liver, heart, muscle, kidney and pancreas functions (Figures S2 and S3). No any abnormal pathological phenotype was observed by the histology analysis of heart, liver and kidney between the control and 7, 8-DHF monkeys (Fig. 2B–G). The animals were found to maintain body weight throughout the 7, 8-DHF treatment period (Fig. 2H). Three months after the first administration (from Day 30 to Day 114), no significant differences between two groups were found in respect to body weight (Fig. 2H). However, it is noteworthy that the weights of control monkeys are significantly higher than those of 7, 8-DHF monkeys at the 11th month (Fig. 2H), implying that long-term administration of 7, 8-DHF may prevent development of obesity.

### Neuroprotective effects of 7, 8-DHF on dopaminergic neurons in MPP^+^-treated monkeys

To induce parkinsonism in cynomolgus monkeys, a quantitative approach was used to develop parkinsonian monkeys with intracerebroventricular injections of MPP^+^, as previously reported to specifically damage brain dopaminergic neurons^11^. We first administered low-dose (0.05 mg each monkey) MPP^+^ twice a week from the 1.5th month and then increased every one or two months up to 0.18 mg at the 9th month (Fig. 2A). For 7, 8-DHF group monkeys, 7, 8-DHF at the dose of 30 mg/kg/day were orally administrated from Day 30, two weeks earlier than MPP^+^ injections, to Day 290 (Fig. 2A).

**Figure 3:**
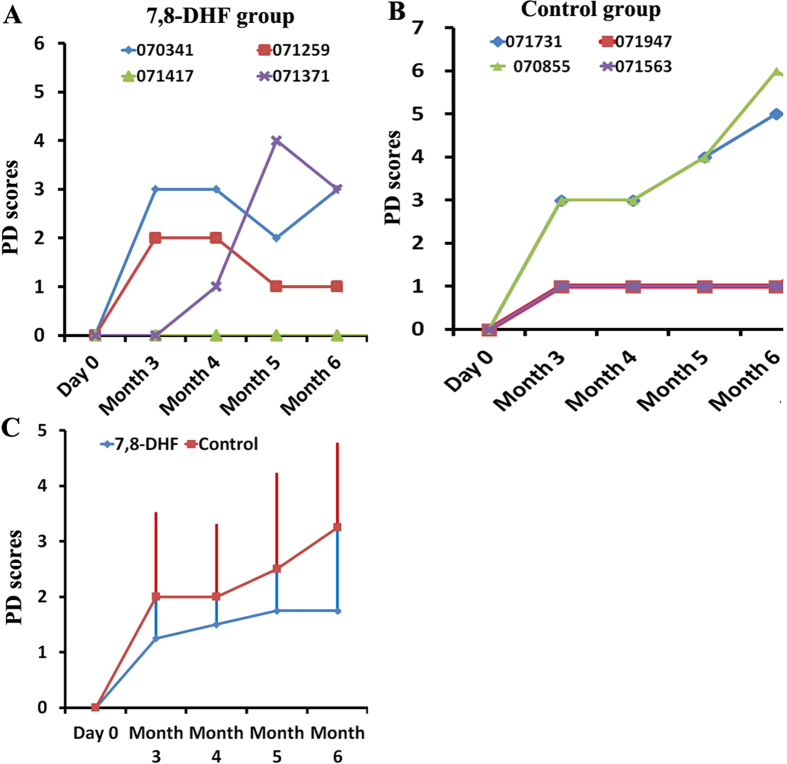
Changes of PD score over the whole modeling and 7, 8-DHF treatment processes in the monkeys. The seven items of PD score were from the Kurlan scale. (**A**) PD scores in four 7, 8-DHF treated monkeys over time. (**B**) PD scores in four control monkeys over time. (**C**) The average distribution of PD symptoms in the two groups. Data are shown as mean ± SD (n = 4 monkeys).

To evaluate monkey behaviors induced by MPP^+^, video recordings of monkeys’ behavior were collected and evaluated for Parkinsonian symptoms using Kurlan scale, which utilizes seven important measures of PD^11^,^14^. By analyzing PD scores, we found that compared to control group (no 7, 8-DHF treatment), 7, 8-DHF treatment prevented the increase of PD scores (Fig. 3A–C). However, different monkeys displayed significant varieties between individuals (Fig. 3A,B). Of note, in the control group, two monkeys (070855 and 071731) displayed parkinsonian symptoms, such as weaker gross motor skills and abnormal gait, furthermore, their parkinsonian symptoms gradually increased over time (Fig. 3B).

**Figure 4:**
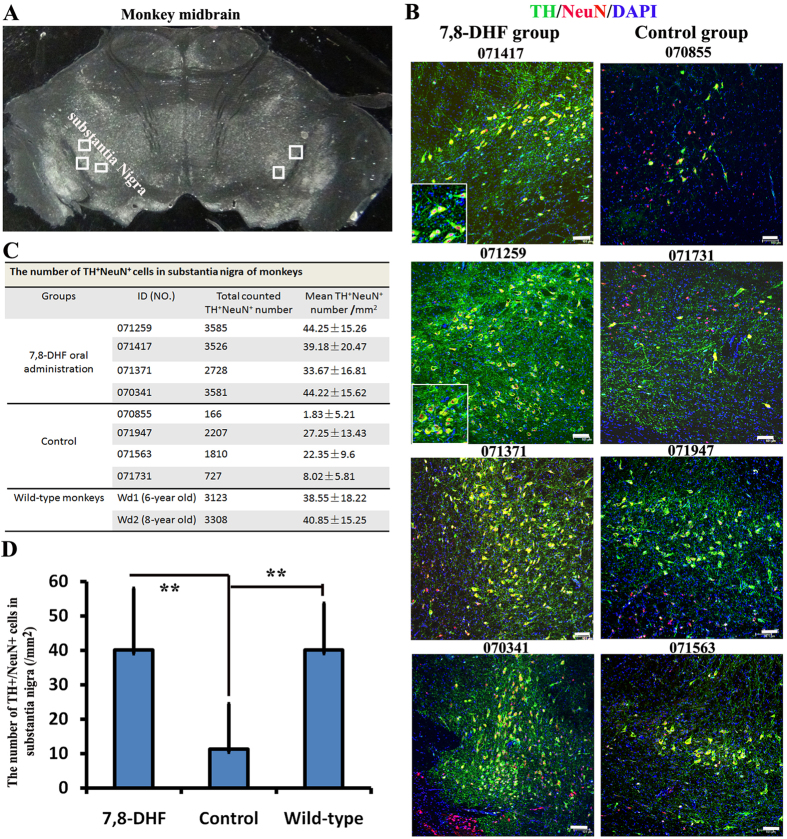
Neuroprotective effects of 7, 8-DHF on dopaminergic neurons in MPP^+^-treated monkeys. (**A**) One representative monkey midbrain under a bright field. Boxes indicate the regions in substantia nigra pars compacta (SNc) that we took photos for counting. (**B**) Representative figures of TH^+^NeuN^+^ dopaminergic neurons in SNc from control and 7, 8-DHF group monkeys. (**C**) Quantifications of TH^+^NeuN^+^ dopaminergic neurons in each SNc of monkeys from control, 7, 8-DHF group and wild-type monkeys. Data are shown as mean ± SD. (**D**) The average number of TH^+^NeuN^+^ dopaminergic neurons for control, 7, 8-DHF group and wild-type monkeys, respectively. Data are shown as mean ± SD (7, 8-DHF for 4 monkeys; Control for 4 monkeys; Wild-type for 2 monkeys). **P < 0.01 by Student’s t test. Scale bars: 100 μm.

To examine neuroprotective effects of 7, 8-DHF, extensive postmortem examinations of survival dopaminergic neurons in SNc were performed on Day 350–380 (Fig. 2A). Two normal monkeys without any treatment were used as wild-type control. Microscopic imaging and stereological cell counts showed more TH^+^/NeuN^+^ dopaminergic neurons of SNc were observed in the 7, 8-DHF than in the control monkeys (Fig. 4A). Consistent with PD scores, in two (070855 and 071731) SNc of four control monkeys, more than 95% and 80% of dopaminergic neurons were lost as compared to 7, 8-DHF and wild-type monkeys, respectively (Fig. 4A–C). About 30% and 44% of dopaminergic neurons were lost in the other two monkeys (071947 and 071563), respectively (Fig. 4A–C). In contrast, the statistical analysis showed that there is no significant difference (P > 0.05) of survival dopaminergic neurons between in 7, 8-DHF and wild-type monkeys (Fig. 4C,D). Together, these data prove strong neuroprotective effects of 7, 8-DHF on monkey dopaminergic neurons in the face of MPP^+^ induced neurotoxicity.

### The protective effects of 7, 8-DHF on dopaminergic neurons are not correlated with astrocyte and microglial activation

**Figure 5:**
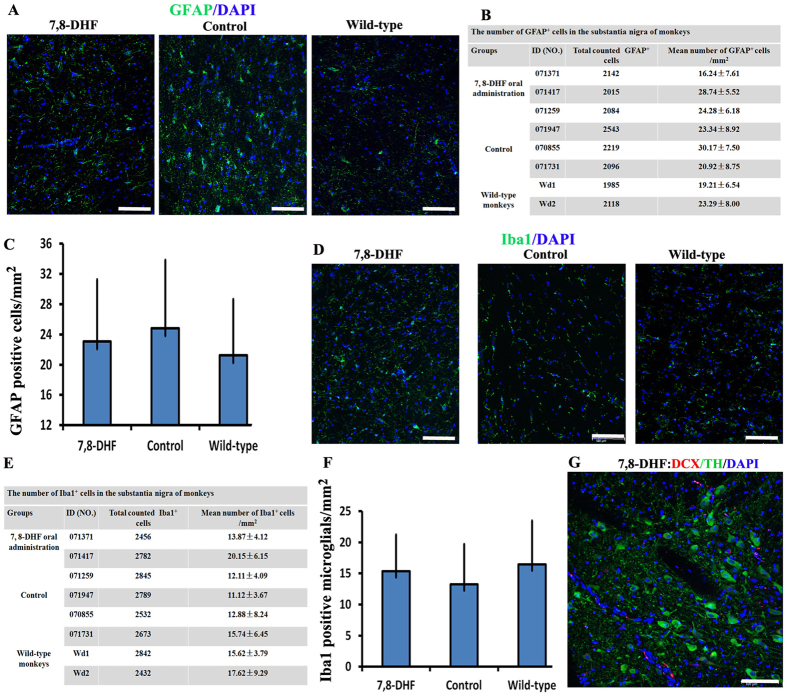
The neuroprotective effect of 7, 8-DHF are not correlated with astrocyte and microglial activation. (**A**) Representative figures of GFAP^+^ astrocytes in substantia nigra pars compacta (SNc) from control, 7, 8-DHF group and wild-type monkeys. (**B**) Quantification of GFAP^+^ astrocytes in different SNc of monkeys. Data are shown as mean ± SD. (**C**) The average number of GFAP^+^ astrocytes for three group monkeys, respectively. Data are shown as mean ± SD (7, 8-DHF for 3 monkeys; control for 3 monkeys; wild-type for 2 monkeys). P > 0.05 by Student’s t test. (**D**) Representative figures of Iba1^+^ microglial in SNc from control, 7, 8-DHF group and wild-type monkeys. (**E**) Quantification of Iba1^+^ microglials in different SNc of monkeys. Data are shown as mean ± SD. (**F**) The average number of Iba1^+^ microglials for three group monkeys, respectively. Data are shown as mean ± SD (7, 8-DHF for 3 monkeys; control for 3 monkeys; wild-type for 2 monkeys). P > 0.05 by Student’s t test. Scale Bars: 100 μm (**G**) No any DCX+ new neuron was generated in substantia nigra pars compacta (SNc) from 7, 8-DHF group monkeys.

Previous studies showed that chronic inflammation is a major characteristic feature of PD. Microglia and astrocytes are known to mediate chronic inflammation, and their activations result in the degeneration of dopaminergic neurons^15^. To evaluate whether 7, 8-DHF protected dopaminergic neurons from apoptosis by inhibiting the chronic inflammation or glial proliferation, we also analyzed the number of microglia and astrocytes in slides. The data showed that 7, 8-DHF treatment failed to result in abnormal change of the microglia and astrocytes (Fig. 5A–F). We also used Doublecortin (DCX) staining to evaluate whether new neurons were generated in 7, 8-DHF group monkeys. Occurrence of neurogenesis was not observed in the SNc (Fig. 5G). Together, these data demonstrated that 7, 8-DHF maintains the number of dopaminergic neurons by direct neuroprotective effects.

## Discussion

Here, our results in the present study demonstrate that 7, 8-DHF displays remarkable neurotrophic actions by protecting dopaminergic neurons from apoptosis in the face of MPP^+^ induced neurotoxicity in monkeys. In mouse assays, 7, 8-DHF has been extensively demonstrated the ability to mimic BDNF and exert a variety of neurological actions in numerous models including PD^5^, stroke^5^, Alzheimer’s Disease^16^, Huntington’s disease^17^, depression^18^,^19^. axon regeneration^20^, learning and memory^21^. Evidently, rodent models are of great importance in disease study and in safety and function assays of candidate drugs, but they may have considerable limitations in neuronal circuitry, behavior, learning and memory^22^. Due to their genetic proximity to humans and their highly developed social skills, non-human primates (NHPs) are certainly without these problems when used, suggesting that NHPs are extremely valuable as experimental animal models^23^. Our study is the first time to demonstrate that 7, 8-DHF treatments can attenuate dopamingeric neuron loss in higher NHPs, implying that this promising small molecule may be transited into a clinical useful pharmacological agent.

Impairment of the BDNF-TrkB pathway has long been believed to have an essential role in PD pathogenesis. Accumulating evidences support that BDNF provides a novel therapeutic strategy for PD treatment. Nevertheless, BDNF possesses the intrinsic drawbacks as a pharmacological agent, including *in vivo* instability and poor pharmacokinetics. Previous studies showed that 7, 8-DHF is a TrkB agonist that can cross the blood–brain barrier. However, the safety of pharmaceuticals needs to be assessed using non-rodent models because these tests are carried out with the aim of protecting human patients in clinical trials. The half-life of 7, 8-DHF in monkey plasma is about 4–8 hrs, longer than about 134 mins in mouse after oral gavage of the drug^13^, implying that NHPs and rodent may have different pharmacodynamic responses. Further studies need to address potential mechanisms. Importantly, long-term (seven months) treatment with 7, 8-DHF reveals no demonstrable toxicity at a dose of 30 mg/kg, a 6-fold higher than mouse (5 mg/kg), implying that the small molecule may be high safe as a potential clinical pharmacological agent.

For both sporadic and familial PD patients, motor symptoms appear when >50% of dopaminergic neurons in the substantia nigra have degenerated^24^. As expected, the two monkeys (071947 and 071563) displayed no abnormal phenotype in presence of more than 50% dopamine neurons. In contrast, 070855 and 071731 monkeys displayed PD symptoms, such as weaker gross motor skills and abnormal gait when more than 95% and 80% of dopaminergic neurons were lost, consistent with the previous report^11^.

In summary, our study shows neuroprotective effects of 7, 8-DHF on dopaminergic neurons in the monkeys. However, one rather important question need to be demonstrated whether 7, 8-DHF have cure effects for PD monkey models in the future.

## Materials and Methods

### Animals and ethics statement

The monkey care and experimental protocol was approved by the Ethics Committee of Yunnan Key Laboratory of Primate Biomedical Research and Kunming Biomed International (AAALAC accredited). The monkeys have been maintained in indoor facilities and all methods were carried out in accordance with the National Institute of Health Guide for the Care and Use of Laboratory Animals and the guidelines of American Association for Accreditation of Laboratory Animal Care (AAALAC). Eight health cynomolgus monkeys (Macaca fascicularis) with specific pathogen free (SPF) quality were used in this research and randomly subdivided into two groups (Fig. 1A). These monkeys are negative of various kinds of viruses (B virus, measles virus, simian varicella virus, simian immunodeficiency virus, simian T cell leukemia virus, simian D type retrovirus, simian cytomegalovirus, simian Epstein-Barr virus, and simian foamy virus), bacteria (Shigella, Salmonella and Mycobacteria spp.) and intestinal helminth. These monkeys were not used for any other experiments before. Two health monkeys, who never participated in any experiments, were sacrificed for pathological examinations as wild-type control. The detailed information of monkeys used in the study is shown in Fig. 1A. Each monkey was alone housed in one cage with the USA standard, respectively. The light/dark cycle for these monkeys is 12 h/12 h. The monkey housing air cleanliness, temperature and humidity were strictly controlled following the Guidance of AAALAC.

### Surgery and intracerebroventricular administration of 1-methyl-4-phenylpyridinium (MPP^+^)

All operations and treatments followed the reported protocol^11^ and were in strict accordance with ethical requirements of animal research^25^. Briefly, an intubation operation of the left lateral ventricle was performed, as described below, in eight monkeys prior to MPP^+^ (D048, Sigma, USA) injections. During surgery, monkeys were injected intramuscularly with atropine (0.5 mg/ml, 1 ml), followed by an intramuscular injection of ketamine (0.15 g/ml, 0.5–0.7 ml) and then, 10 min later, with sodium pentobarbital (40 mg/ml, 20 mg/kg) during the operation. After anesthesia, the monkey’s head was fixed into the stereotaxic instrument. The animals’ health was monitored throughout the surgery by a veterinarian and an unconsciousness state was maintained. The coordinates of left ventricle of the cynomolgus monkey were located at 23 mm in front of the A/P zero location, 1.5 mm left to the median sagittal line, and 15–19 mm beneath the skull. A hole in the skull was drilled at these coordinates. A sterile pipe made of stain-less steel (21 G, OD = 0.813 mm, ID = 0.584 mm) was inserted vertically into the ventricle confirmed by cerebrospinal fluid spilling from the pipe. Then, the implanted pipe was filled with a solid removable plug. The implanted pipe was protected by a dental cement shell, which was fixed on the skull with 3–4 titanium screws. A protective cap was fixed into the dental cement as a cover. Post-operative monkeys were trained to sit in monkey chairs for administration of MPP^+^ without anesthesia. 100 μl of MPP^+^ were injected in the lateral ventricle with a syringe via a plastic hose as described^11^. The MPP^+^ doses were gradually increased from 0.05 mg to 0.18 mg/monkey (Fig. 2A). MPP^+^ was delivered into the lateral ventricle twice a week.

### Oral administration of 7, 8-DHF

7, 8-DHF was purchased from TOKYO CHEMICAL INDUSTRY CO., LTD (D1916). The dose of 30 mg/kg for each monkey was embedded in the bananas or apple and was orally administrated at 8:00am every day.

### Analysis of 7, 8-Dihydroxyisoflavone and its two metabolites in monkey plasma

Each monkey was subjected to one blood draw at 0.5-, 1.5-, 2.0-, 4.0-, 8.0- and 24- hours after oral administration, respectively. For fasting, food but not water was withheld for at least 12 h. Following fasting, each monkey was weighed and the dose was calculated according to body weight. After administration of 7, 8-DHF, blood was collected through venous. Blood aliquots (500–1000 μl) were collected in tubes coated with sodium heparin, mixed gently, kept on ice and centrifuged at 2,500 *g* for 15 min at 4 °C within 1 h. The plasma was harvested and kept frozen at −80 °C until further processing. Before examination of 7, 8-DHF and its metabolites, 7, 8-DHF group monkeys have been treated by 7, 8-DHF for two months. Analysis of 7, 8-DHF and its two metabolites in monkey plasma were performed by HPLC-MS/MS system. The detailed information was described in Supplemental Experimental Procedures.

### Behavioral data recording

Video recordings of monkeys’ behavior were collected once a week (Wednesday afternoon between 14:00 and 15:00) on digital cameras and evaluated for parkinsonian symptoms using part A of the Kurlan scale, which has been widely accepted as a valued scale for PD research in Old World Monkeys^11^,^14^,^26^. The video recorded data from control group and 7, 8-DHF group were blindly analyzed by 2–3 observers, who did not know any information of each video recording, such as recording time and its relative experiment group.

### Evaluation of general health state

Physiological and biochemical parameters presage of hematology, liver, heart, muscle, kidney and pancreas functions were examined by following human clinical testing methods.

### Pathological examinations and tissue analysis

Monkeys were euthanized (1 ml ketamine, 0.15 g/ml, i.m.) for perfusion with 500 ml saline and 500 ml 10% formaldehyde in PBS. The brains were removed and fixed again in formalin for 3–4 days, and then equilibrated with 20% and 30% sucrose. Coronal sections of the substantia nigra were obtained by sectioning the tissue on a freezing microtome (Leica, CM1850UV-1-1). Slice thickness was set at 18 μm. Slices were first blocked with 10% sheep serum for 45 min at room temperature and then incubated with primary antibody overnight at 4 °C. Primary antibodies are listed in the following: NeuN (Neuronal Nuclei) (Millipore, MAB377, 1:100); Iba1 (Wako Pure Chemical Industries Ltd, 019–19741, 1:300); Tyrosine Hydroxylase (TH) (Millipore, AB152, 1:400); Doublecortin (DCX) (Millipore, MABN707, 1:200); GFAP (Sigma, G9269, 1:2000). The following day, cells were washed three times with PBS media and incubated with Alexa 488 or Rhodamine-conjugated secondary antibodies (Invitrogen: goat-anti-rabbit, goat-anti-mouse, donkey- anti-goat, donkey-anti-chicken, 600×) in PBS for one hour at RT. Nuclei were visualized with DAPI staining (Sigma-Aldrich).

### Statistical analysis

To accurately count cell numbers, every 10th slide from anterior to posterior of the substantia nigra tissues was used to stain TH, NeuN, Iba1 and GFAP, respectively. The five different regions every slide were taken using 10x magnification (objective) under the Leica SP8 confocal microscopy, respectively (Fig. 4A). The five regions were fixed to all monkeys including wild-type, control group and 7, 8-DHF group. About 20 slides were used to count positive cells every monkey. Morphologically intact cell with a nucleus was counted. All of cell count results were converted into cell number/mm^2^. Quantification data were represented as mean ± standard deviation (s.t.d) using Microsoft Excel STDEV Function. The significance difference between two samples was evaluated by the unpaired two-sample Student’s *t*-test using Excel software. P < 0.05 was considered as statistical significant differences.

## Additional Information

**How to cite this article**: He, J. *et al.* Neuroprotective Effects of 7, 8-dihydroxyflavone on Midbrain Dopaminergic Neurons in MPP^+^-treated Monkeys. *Sci. Rep.* **6**, 34339; doi: 10.1038/srep34339 (2016).

## Supplementary Material

Supplementary Information
