# Supplementary material for: Neuroprotective Effects of 7, 8-dihydroxyflavone on Midbrain Dopaminergic Neurons in MPP+-treated Monkeys
Source: Sci Rep. 2016 Oct 12;6:34339. doi: 10.1038/srep34339 (PMC5059638; doi:10.1038/srep34339)
Supplement: Supplementary Information [file srep34339-s1.doc]

**Neuroprotective Effects of 7, 8-dihydroxyflavone on**

**Midbrain Dopaminergic Neurons in MPP^+^-treated Monkeys**

Jingjing He^1^, Zheng Xiang^1^, Xiaoqing Zhu^1^, Zongyong Ai^1^, Jingsong Shen^1^, Tianzhuang Huang^1^, Liegang Liu^2^, Weizhi Ji^1^, Tianqing Li^1*^

1. Yunnan Key Laboratory of Primate Biomedical Research；Institute of Primate Translational Medicine, Kunming University of Science and Technology, Kunming, 650500, Yunnan, China
2. Department of Nutrition and Food Hygiene, Hubei Key Laboratory of Food Nutrition and Safety,  School of Public Health, Tongji Medical College, Huazhong University of Science and Technology, 13 Hangkong Road, Wuhan 430030, China

*To whom correspondence should be addressed. Email: [litq@lpbr.cn](mailto:litq@lpbr.cn) (T.L.), Tel: 86-871-65952872

**Supplemental Experimental Producers**

**2.1. Chemicals and materials**

7-hydroxy-8-methoxyflavone and 8-hydroxy-7-methoxyflavone were provided by Pathology & Laboratory Medicine, Emory University School of Medicine (USA). The purity of these reference standards was more than 98.0%. Daidzeinas internal standard was obtained from National Institute for Food and Drug Control (Beijing, China). HPLC-grade acetonitrile and methanol were supplied by Thermo Fisher Scientific Company (USA). HPLC-grade formic acid,ammonium acetate and acetic acidwas from Sigma-Fluka Company(USA). β-Glucuronidase (type HP-2, from Helix pomatia,≥100000 units/ml) which also has sulfatase activity was supplied by Sigma (Company Inc, Saint Louis, USA). All other reagents were of analytical grade.

**2.2. LC-MS/MS instrumentation and analytical condition**

Agilent 1200 HPLC system equipped with G1312B [Binary Pump](https://www.google.com/url?sa=t&rct=j&q=&esrc=s&source=web&cd=3&cad=rja&uact=8&ved=0CCkQFjAC&url=https%3A%2F%2Fconquerscientific.com%2Fproduct%2Fagilent-1200-series-binary-pump-sl%2F&ei=3GaOVPvcCYOlgwSW7YC4BA&usg=AFQjCNGDlGKGwRnnTfOsjzEPrw1EDS8MNA&sig2=KYUKuXSuu8DZEMkiHYIRAw), G1379B vacuum degasser, G1317D Hip-ALS autosampler and G1316B thermostatted column compartment and Agilent 6460 triple quadrupole mass spectrometer equipped with electrospray ionization (ESI) were used for all analyses. All the operations, the acquiring and analysis of data were performed by Mass Hunter software (Agilent Corporation, MA, USA).

The chromatographic separation of 7, 8-DHF and 4, 7-DHF (IS) was performed on a BETASIL Phenyl Column (2.1mm×150mm, 3um, Thermo Scientific, USA) . The column was maintained at 35 ◦C and the injection volume was 5uL.The mobile phase consisted of acetonitrile and 0.1‰ aqueous formic acid using isocratic elution (80% acetonitrile for 7, 8-DHF, 32% acetonitrile for 7-hydroxy-8-methoxyflavone and 8-hydroxy-7-methoxyflavone) and was delivered at a flow rate of 0.3 mL/min.

Ionization was achieved using electrospray in the negative mode with the spray voltage set at 4000V. Nitrogen was used as nebulizer gas and nebulizer pressure was set at 45psi with a source temperature of 37.5^◦^C. Desolvation gas (nitrogen) was heated to 3.5^◦^C and delivered at a flow rate of 10 L/min. High purity nitrogen was employed as the collision gas at a pressure of approximately 0.1 MPa. The analytes were determined by monitoring the precursor–product combination in multiple reaction monitoring (MRM) mode and quantitative parameters are listed in Table 1.

Table1. List of selected MRM parameters, fragmenter voltage (FV), and collision energy (CE) for each analyte and IS.

| Analyte*m*/*z* of precursor ion(Da) *m*/*z* of product ion(Da) FV (V) CE (eV) |
| --- |
| | 7,8-Dihydroxyisoflavone 253 123120 30  7-hydroxy-8-methoxyflavone 267.1 252 80 10  8-hydroxy-7-methoxyflavone 267.125 80 10  Daidzein (IS) 253132140 36 | | --- | |

**2.3. Sample preparation**

**2.3.1. Determination of free 7,8- Dihydroxyflavone and its two metabolites in monkey plasma**

A 100uL plasma sample was spiked with 20 uL IS and 20 uL methanol. The mixture was vortex mixed for 1 min and then 300uL methanol containing 0.1% formic acid was added. After vortex mixing vigorously for 2 min and centrifuging at 14 000 rpm atroom temperature for 10 min, the supernatant was transferred to another vial and evaporated to dryness at 45℃ in a vacuum concentration system.The residue was reconstituted in 100 uL acetonitrile and centrifuged at 14 000 rpm at room temperature for 10 min. At last, 5uL aliquots of the supernatant were injected into the HPLC-MS/MS system.

Plasma samples, which concentrations were greater thanthe upper limit of the calibration curve, could be reanalyzedby appropriate dilution.

**2.3.2. Determination of total 7, 8- Dihydroxyflavone and its two metabolites in monkey plasma**

Sample plasma (100 uL) was incubated overnight with 100uL of enzyme (β-Glucuronidase≥400 units/mL in 0.5 mol/L ammonium acetate buffer (pH 5.0)) at 37℃. After cooling to the room temperature, the enzymehydrolyzed samples were added with 20 uL of IS and20 uL methanol. Then the following processing was thesame as described above.

**2.4. Preparation of calibration standards and quality control (QC)samples**

The standard stock solutions of 7, 8-DHF, 7-hydroxy-8-methoxyflavone and 8-hydroxy-7-methoxyflavone were separately prepared in methanol at concentrations of 100 ug/mL. The stock solution was further diluted with methanol to achieve a series of mixed working standard solutions with concentrations 2-1000 ng/mL for each analyte. The working solution of IS (500ng/mL) was also prepared by diluting 4,7-DHF with methanol.

Then assay standard samples were prepared by spiking 100uL blank monkey plasma with standard mixture working solutions (20uL) and IS working solution (20uL), in which the final concentrations were 4, 10, 20, 40,100 and 200 ng/mL for 7, 8-DHF,0.4, 2, 4, 10, 20, 40 and 100 ng/mL for 7-hydroxy-8-methoxyflavone and 0.5, 2, 4, 10, 20, 40 and 100 ng/mL for 8-hydroxy-7-methoxyflavone.The Quality control (QC) samples were separately prepared at three concentrations containing 7, 8-DHF (4, 20 and 100 ng/mL), 7-hydroxy-8-methoxyflavone (2, 10 and 40 ng/mL) and 8-hydroxy-7-methoxyflavone (2,10 and 40 ng/mL) in the same fashion. All the solutions were kept at 4^◦^C.

Supplemental Figures


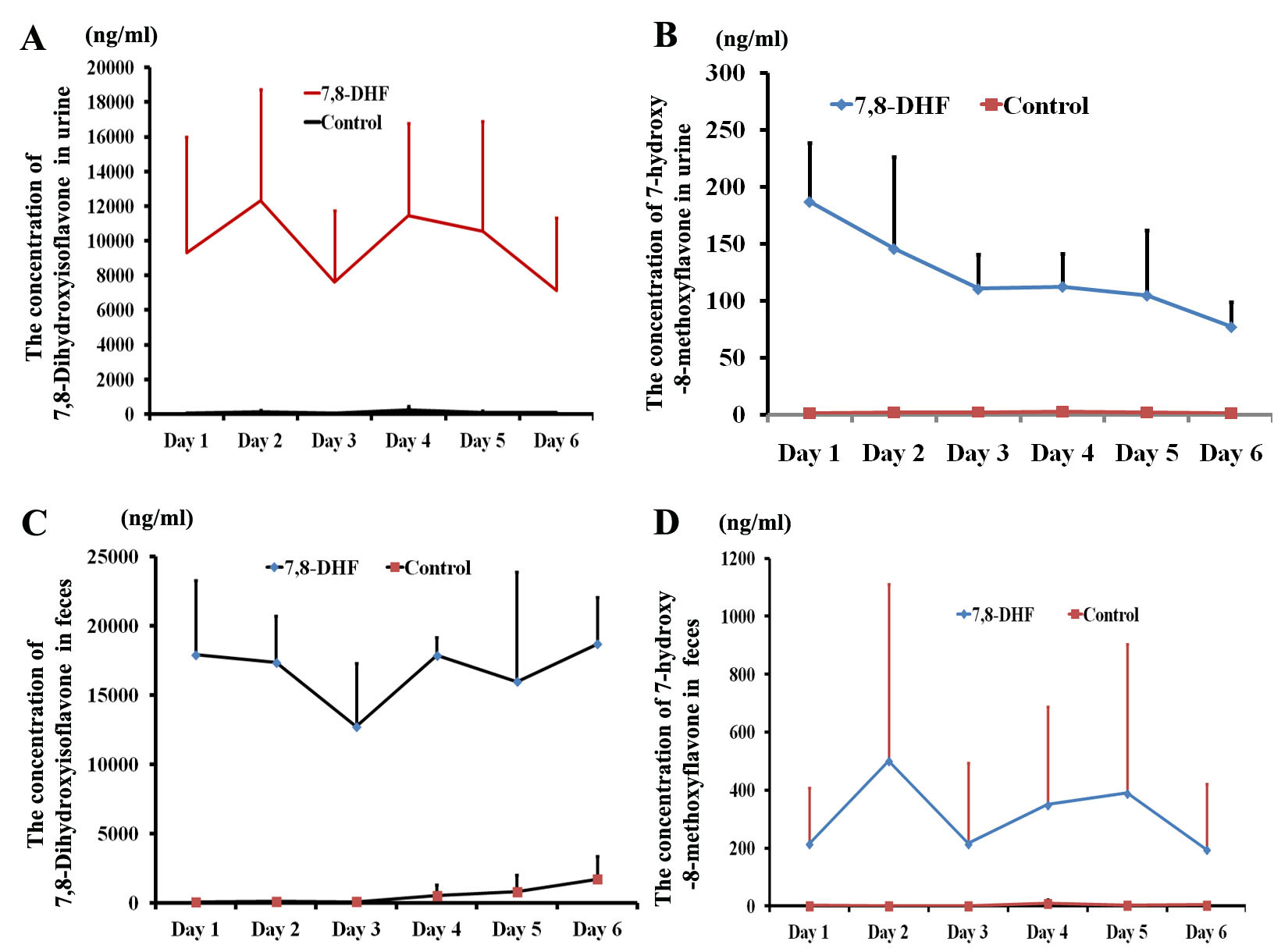


Figure S1. *In vivo* pharmacokinetics of 7, 8-dihydroxyflavone (7, 8-DHF) in monkeys, relative to Figure 1. (A) The concentration of 7, 8-DHF in monkey urine from consecutive six nights after oral administration. (B) The concentration of the matebolite 7-hydroxy-8-methoxyflavone in monkey urine. (C) The concentration of 7, 8-DHF in monkey feces from consecutive six nights after oral administration. (D) The concentration of the matebolite 7-hydroxy-8-methoxyflavone in monkey feces. The examination was performed in the condition absence of β-Glucuronidase.


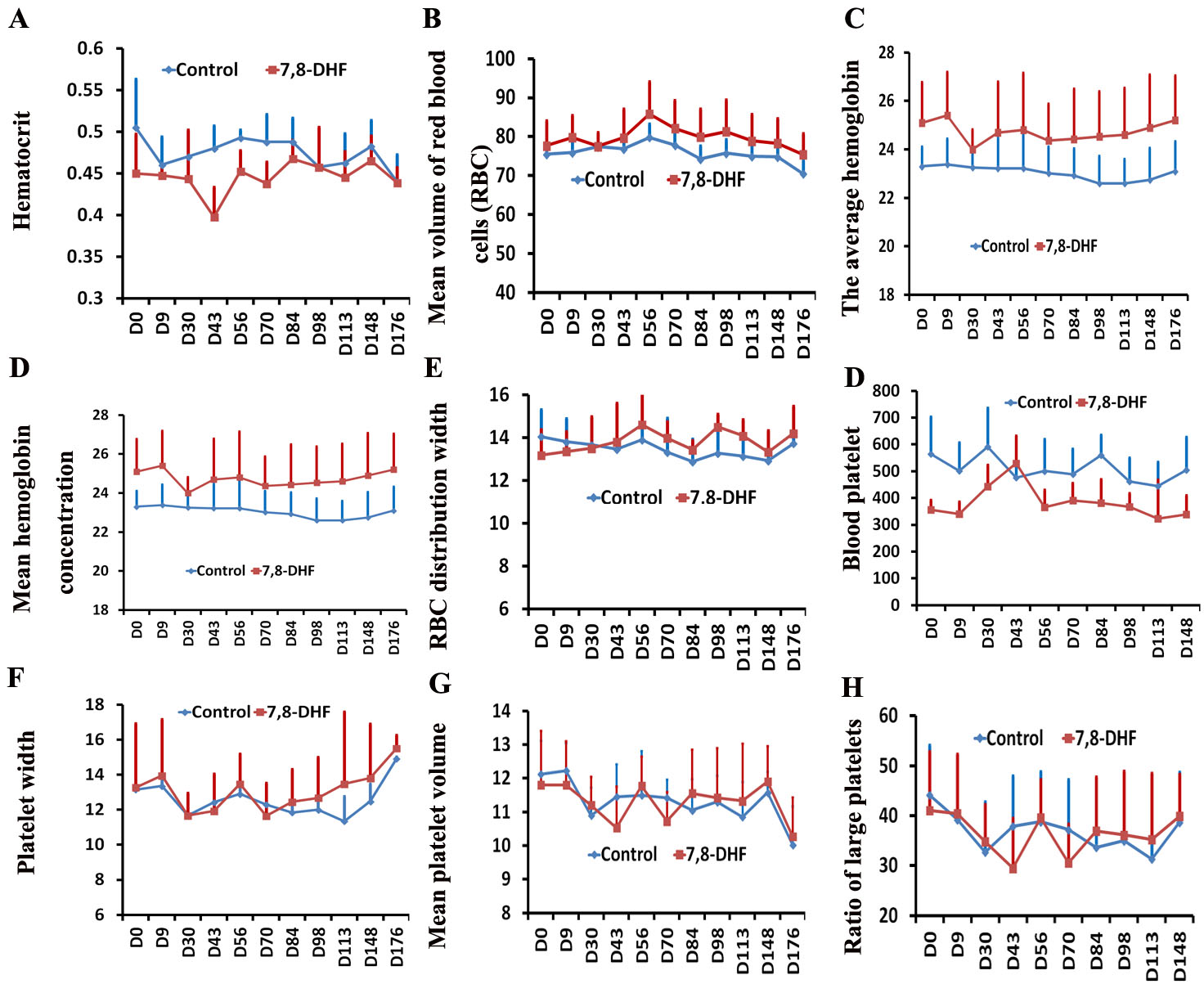


Figure S2. Long-term oral administration of 7, 8-DHF is safe for monkeys, relative to Figure 2. The hematology in control group (with only MPP+ treatment) and 7, 8-DHF group (accompanying with MPP+ treatment) monkeys. Data are shown as mean ± SD (n=4 monkeys).


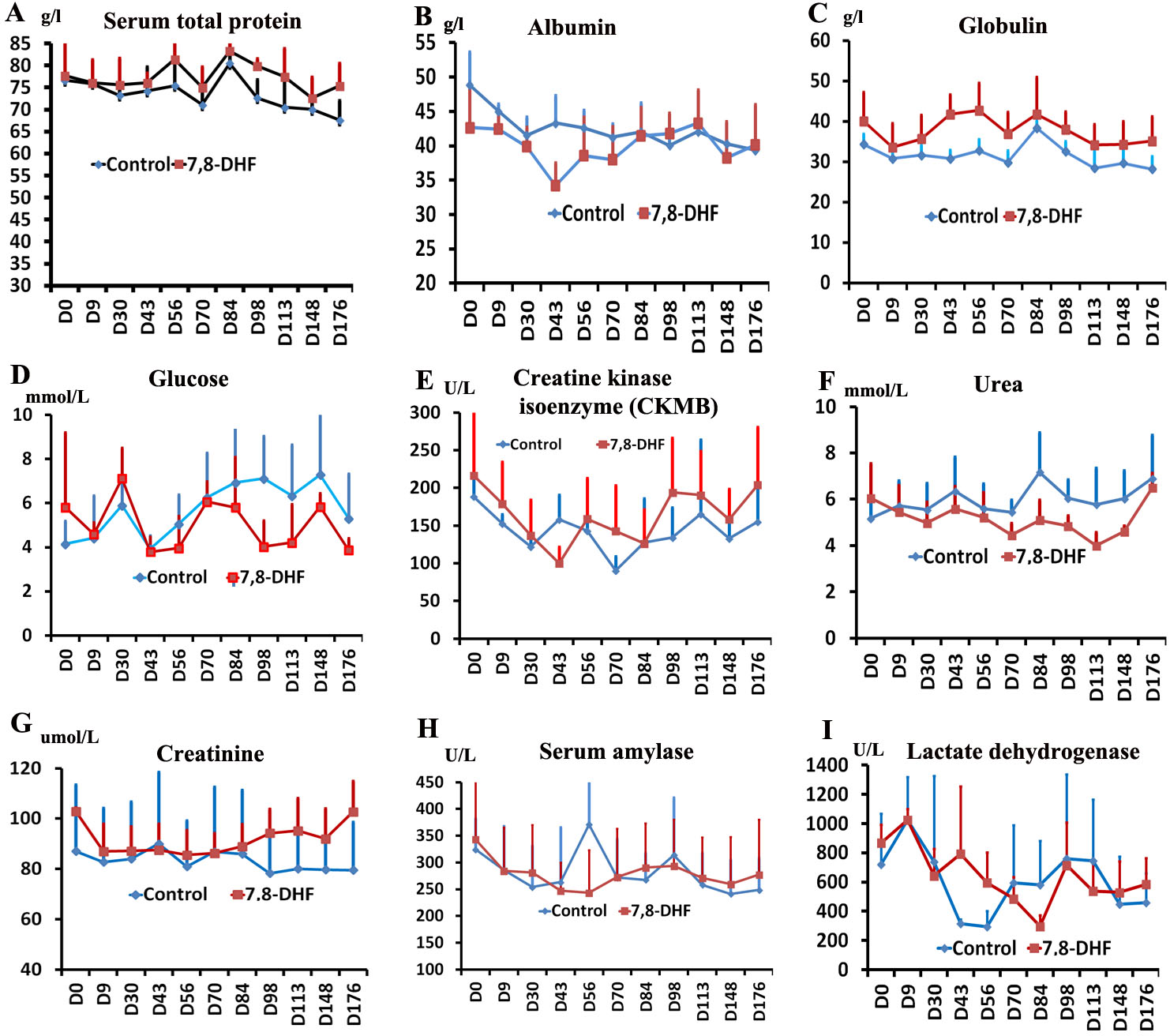


Figure S3. Long-term oral administration of 7, 8-DHF is safe for monkeys, relative to Figure 2. The Liver function, heart function and kidney function for control group and 7, 8-DHF group monkeys over time. Data are shown as mean ± SD (n=4 monkeys).
